# An Energy-Aware Routing Protocol for Query-Based Applications in Wireless Sensor Networks

**DOI:** 10.1155/2014/359897

**Published:** 2014-02-13

**Authors:** Ehsan Ahvar, Shohreh Ahvar, Gyu Myoung Lee, Noel Crespi

**Affiliations:** ^1^Wireless Networks and Multimedia Services Department, Institut Mines-Telecom, Telecom SudParis, 9 rue Charles Fourier, 91011 Evry Cedex, France; ^2^Electrical and Computer Engineering Department, Isfahan University of Technology, Isfahan, Iran

## Abstract

Wireless sensor network (WSN) typically has energy consumption restriction. Designing energy-aware routing protocol can significantly reduce energy consumption in WSNs. Energy-aware routing protocols can be classified into two categories, energy savers and energy balancers. Energy saving protocols are used to minimize the overall energy consumed by a WSN, while energy balancing protocols attempt to efficiently distribute the consumption of energy throughout the network. In general terms, energy saving protocols are not necessarily good at balancing energy consumption and energy balancing protocols are not always good at reducing energy consumption. In this paper, we propose an energy-aware routing protocol (ERP) for query-based applications in WSNs, which offers a good trade-off between traditional energy balancing and energy saving objectives and supports a soft real time packet delivery. This is achieved by means of fuzzy sets and learning automata techniques along with zonal broadcasting to decrease total energy consumption.

## 1. Introduction

A wireless sensor network (WSN) is composed of a large number of sensor nodes, which are densely deployed either inside the phenomenon or very close to it. Since the sensor nodes are often inaccessible, the lifetime of a sensor network depends on the lifetime of the power resources of the nodes. Power is also a scarce resource due to the size limitations [1].

Many routing algorithms and protocols have been proposed for different types of WSNs (i.e., [2–4]) among which we have identified a category known as query-based routing. In query-based routing protocols, a station *S* sends queries to find specific events among the WSNs. The strategies used for routing these queries and their corresponding replies can be classified into two major groups, energy savers and energy balancers. The former tries to decrease the energy consumption of the network as a whole and thereby increase the operation lifetime which also usually leads to the utilization of the shortest paths. The latter, on the other hand, tries to balance the energy consumption of the nodes to prevent partitioning of the network.

Rumor is an energy saving protocol that provides an efficient mechanism combining push and pull strategies to obtain the desired information from the network [5]. In Rumor, the nodes generating events send notifications that leave a sticky trail along the network. When query agents visit a node where an event notification agent has already passed through, they can find pointers (i.e., the trail) towards the location of the corresponding source. In general terms, when a node receives a query, two things can happen: (i) the node already has a route toward the target event, so it only needs to forward the query along the route; or (ii) the node does not have a route, and therefore, it forwards the query to a random neighbor. The random selection of the neighbor in this case is relatively constrained, since each node keeps a list of recently visited neighbors to avoid repeatedly visiting them. Clearly, the forwarding strategy in Rumor could end up producing spiral paths, so an intuitive improvement would be to reduce its level of routing indirection. To this end, Chou et al. proposed the straight line routing (SLR) protocol [6], which aims at making the routing path grow as straight as possible. More recently, Shokrzadeh et al. have made significant efforts to improve Rumor in different aspects with their directional Rumor (DRumor) [7]. Shokrzadeh et al. later improved their DRumor protocol by means of what they called the second layer routing (SecondLR) [8]. SecondLR uses geographical routing immediately after locating the source of an event, and Shokrzadeh et al. have shown that this approach considerably improves the performance of DRumor. Despite these efforts, current query-based routing protocols are mainly energy savers and have shown relatively poor performance when it comes to balancing energy consumption. Much more recently, Ahvar et al. have proposed the energy-aware query-based routing protocol (EQR), an energy saving and balancer routing protocol [9]. EQR uses zonal broadcasting to reduce energy consumption.

This paper presents a routing strategy applicable to various forms of query-based applications that offers a reasonable trade-off between the energy saving and balancing. More precisely, we propose an energy-aware routing protocol for query-based applications in WSNs called the ERP, which is supported by learning automata and fuzzy sets and which uses zonal broadcasting to decrease the total energy consumed. Our initial results demonstrate the potential and the effectiveness of ERP, making it a promising candidate for a number of WSN applications.

The rest of the paper is organized as follows. In Section 2, we introduce our design goals.  Section 3 presents the main contribution of this paper which is basically the ERP routing strategy. The assessment of ERP is covered in Section 4, and Section 5 concludes the paper.

## 2. Design Goals

More specifically, ERP satisfies the following design objectives.

(1) Energy-distance awareness: energy-awareness means both energy saving and energy balancing. Energy saver protocols try to decrease the energy consumption of a network to increase network lifespan. They usually try to find the minimum path length to reduce energy consumption. Energy balancer protocols try to balance the energy consumption of nodes to prevent network partitioning. Finding the best route only based on energy balancing may lead to longer paths with greater delay, and finding the best route based only on energy savings and optimal distance may lead to network partitioning. The ERP is an energy saver and an energy balancer at the same time. It achieves a trade-off between distance and energy by using learning automata and fuzzy sets techniques.

(2) Accuracy: finding the best node as a next hop in aspects of energy saving and balancing is a big challenge for routing protocols. Most of energy-aware routing protocols find the next hop based on only one measurement factor, such as energy level. The ERP, however, considers hop count and distance as well as energy level, simultaneously, utilizing more than one decision-making technique to achieve more exact results.

(3) Localized behavior and scalability: the ability to maintain performance characteristics irrespective of the size of the network is referred to as scalability. Pure localized algorithms are those in which any action invoked by a node should not affect the system as a whole. In these protocols, a node usually uses flooding to discover new paths. In WSNs, where thousands of nodes communicate with each other, broadcast storms may result in significant power consumption and even in a network meltdown. To avoid that situation, most of the distributed operations in ERP are localized to achieve high scalability.

(4) Minimal state architecture: the physical limitations of WSNs, such as large scale, high failure rate, and constrained memory capacity, demand a minimal state approach. The ERP only maintains the immediate neighbor's information and so it does not need a large routing table. Thus, its memory requirements are minimal.

(5) Link failure detection: the ERP has the ability to find a broken link. Unlike most protocols, it does not use the acknowledge packet to check link stability. The ERP verifies links by means of the overhearing technique described herewith.

(6) Minimal control packet: in many routing protocols, the nodes' energy levels are forwarded to neighbors by acknowledge packets. The proposed routing protocol uses the overhearing technique for updating energy levels. In most routing protocols, nodes with very low energy levels send a packet to warn their neighbors. The ERP instead has a threshold, and when each neighbor forwards a packet, all the neighbors receive it and compare the attached energy level of the sender node to the threshold level. If the sender energy is under the threshold, the sender is considered to be a dead node and will not be selected again as a next hop. Therefore, the proposed protocol does not need an extra packet to announce a dead node. The threshold level is the energy required to send a packet.

## 3. Energy-Aware Routing Protocol (ERP)

**Figure 1:**
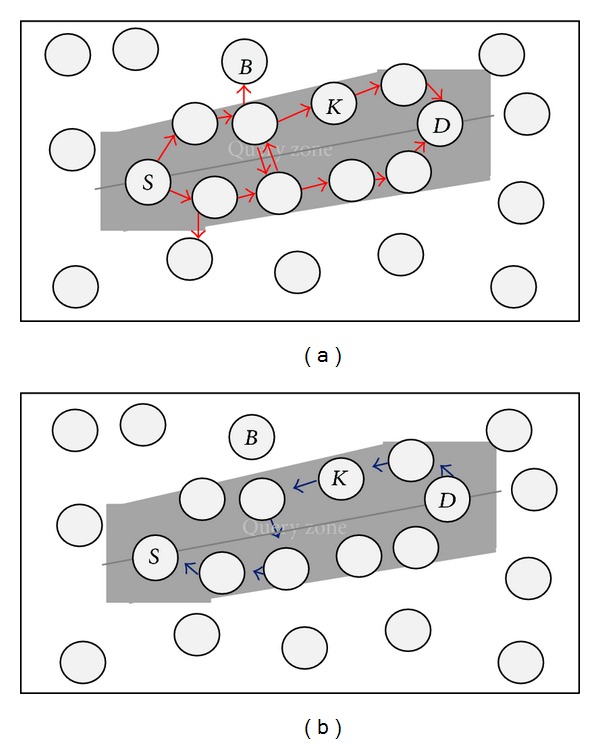
EPR routing mechanism: (a) query broadcasting and (b) data forwarding.

ERP is a query-based routing protocol designed to consider both energy and distance while routing packets across a network. It balances the load among the different sensors with a twofold goal: preventing the sensors from running out of battery while keeping the routes to reach the destinations relatively short. ERP can be divided into two phases: query broadcasting and data forwarding (Figure 1).

### 3.1. Query Broadcasting Phase

When a WSN starts its operation, station *S* that will issue the query packet to find destination *D* will have no zonal information. Therefore, the query mode used by station *S* at the beginning of the operations will typically be a simple broadcasting, which means that the entire region is assumed to be the query zone. The query packet contains the following fields: sender Id (SI), destination Id (DI), sender position (SP1), station position (SP2), destination position (DP), destination radius (DR) and energy level (EN). The SI saves the Id of the query-sender node; the destination Id is carried by the DI field. SP1 and SP2 forward the positions of the sender and the station, respectively, and, if available, DP includes the position of the destination or the center of requested zone. EN indicates the energy level of the sender node. In the first time of query broadcasting for finding destination *D*, the station *S* sets SI, SP2, and EN as its ID, position, and energy level. In this situation, SP1 and SP2 are similar because query sender is the station *S* itself. Also DP and DR fields can be null because in the first time there is no information about location of the destination *D*.

After receiving the query packet, each intermediate node will save information of the query sender (i.e., energy level and also sender and the station position) into its Neighbor List. The components of the Neighbor List for each neighbor are as follows:

 
*Neighbor ID (NID)*: for holding the ID of a neighbor;
 
*Energy Level (EN)*: for holding the energy level of the sender's neighbor;
 
*Hop Count (HP)*: the number of hops from a neighbor to the station;
 
*Sender Position (SP1)*: the position of the sender (neighbor) node;
 
*Station Position (SP2)*: the position of the station;
 
*Module1 (M1)*: the probability associated with a neighbor as computed by the learning automata (LA);
 
*Module2 (M2)*: the membership degree associated with a neighbor as computed by the Fuzzy Set (FS) technique.

After inserting information of the query packet into the Neighbor List, the intermediate node checks the DP field. In first time, the DP field of the received query packet is null and the intermediate node only inserts its ID, position, and energy level and rebroadcasts the query packet. When destination *D* received the query packet, it will respond by sending a data packet (attached some information such as its position) to the station *D*; details of data packet and its routing will be described in Section 3.2.

In the next rounds of query sending, for finding destination *D*, the station *S* has knowledge about previous position of destination *D*. Using this knowledge can help us to limit query zone for reducing energy consumption. Although zone limitation in query broadcasting can reduce energy consumption, using small zone can lead to network partitioning. Because in a small zone just a few nodes transfer all packets and their energy can be finished soon. Therefore, our goal is designing an effective zone that covers important parts of networks such as all neighbors of the station for effective managing of energy near the station.

If we consider that destination *D* at time *t*_0_ was at location *L*, the station *S* at time *t*_1_ computes a circular Expected Domain (ED). For instance, if node *S* knows that node *D* travels with average speed *v* or Velocity, then *S* may assume that the ED is the circular region of radius *v*(*t*_1_ − *t*_0_), centered at location *L* [10]. It means that we expect the destination *D* located at one place inside this domain. Therefore, our query zone must cover this ED.

The radius is computed based on the following:

(1) $$\begin{array}{c} F=\text{Velocity}\times \left(t_{1}-t_{0}\right)+\varepsilon . \end{array}$$

In the equation, Velocity is the average speed. *t*_1_ is the current time and *t*_0_ is the time of the previous location of node *D*. The constant epsilon is used to keep radius nonzero in an immobility status.

Using the ED, the ERP protocol will compute a limited query zone to reduce energy consumption of broadcasting.

**Figure 2:**
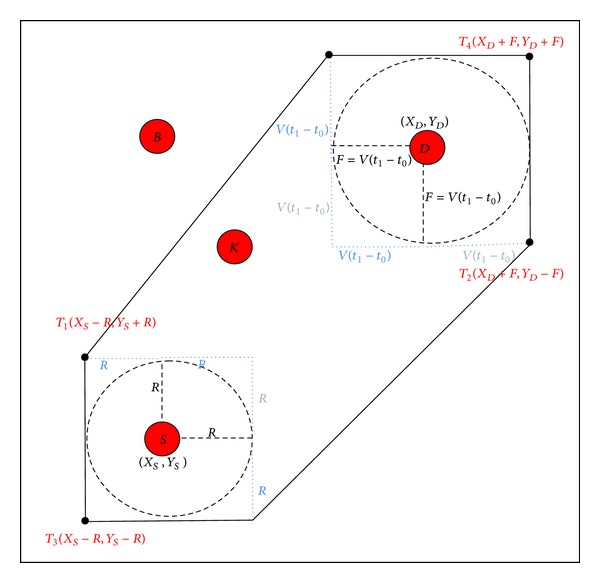
Proposed query zone.

We propose a new query zone with an optimum size (see Figure 2).

The nodes receiving the query packet can forward or discard it depending on their location. For instance, in Figure 2, when node *B* receives the query originally sent from station *S*, it will process the packet but it will not forward it, since *B* is not within the query zone. Instead, node *K* will process and forward the query given that it is inside the zone. In brief, the nodes within the query zone distribute the queries complementing the information originally sent by the station *S* with their own ID, their energy level, the procedure start time, their position, hop count, distance to the station, and a list of hops to prevent forwarding loops. This process is repeated until a destination is found. To determine whether a node *B*(*x*, *y*) is in, on, or outside our zone (the zone can be considered as a positively oriented polygon), the following equation is used:

(2) $$\begin{array}{c} A=\left(x_{2}-x_{1}\right)\left(y-y_{1}\right)-\left(y_{2}-y_{1}\right)\left(x-x_{1}\right). \end{array}$$

If *A* is more than zero for all sides, node *B* is in the zone.

### 3.2. Data Forwarding Phase

When the destination *D* receives the query packet, it replies by sending a data packet. There is a single data packet format for the ERP protocol, which contains the following major fields: Station ID (SI) which is the destination of the data packet or the ID of the station, payload, an array with a size of 5 that saves the 5 last previous hops to prevent loops, energy level (EN) for forwarding the energy level of the data sender node, and sender position (SP) which gives the location of the data sender node as well as the time and destination position files that indicate the location of the destination node at a certain time so that the query zone in the Station can be estimated for a future query.

Procedure of selecting next hop in data forwarding phase is based on learning automata (LA) and fuzzy set (FS) techniques. LA and FS offer their idea about selecting next hop individually and finally one decision maker (DM) algorithm makes final decision.

As we mentioned before, Neighbor List includes M1 and M2 fields. M1 field is filled by learning automata (LA) and M2 field by fuzzy set (FS). Each node that receives a data packet runs its DM algorithm. The DM looks at the Neighbor List and finds the neighbor with highest probability in the M1 field (called ID^(*M*1)^) and the neighbor with highest membership degree in the M2 field (called ID^(*M*2)^). Actually, the highest probability neighbor in the M1 field is a selected neighbor when using the learning automata technique and the highest membership degree in the M2 field is a selected neighbor by means of the FS technique. Note that if the selections made by the LA and FS techniques are the same neighbor, then that neighbor is chosen as the next hop. Otherwise, the node runs a basic sequence of tie-breaking rules until the next hop is selected.

The processing of the DM module is summarized in Algorithm 1. In this algorithm, *ℰ*_ID_^(*M*1)^ and *H*_ID_^(*M*1)^ are the energy level and hop count, respectively, of selected neighbor ID^(*M*1)^ by means of learning automata and *ℰ*_ID_^(*M*2)^ and *H*_ID_^(*M*2)^ are the energy level and hop count, respectively, of selected neighbor ID^(*M*1)^ by utilizing the FS technique.

Method of filling M1 and M2 fields by LA and FS is described in the following.

#### 3.2.1. Membership Degree Computation

The theory of fuzzy sets was introduced by Zadeh in 1965 [11]. Since the pioneering work of Zadeh, there has been a great effort to obtain fuzzy analogues of classical theories. Fuzzy set theory is a powerful tool for modeling uncertainty and for processing vague or subjective information in mathematical models. Their main directions of development have been quite diverse and they have been applied to a great variety of real problems. The notion central to fuzzy systems is that truth values (in fuzzy logic) or membership values (in fuzzy sets) are indicated by a value on the range of [0, 1], with 0 and 1 representing absolute falseness and absolute truth, respectively.

The ERP algorithm considers a fuzzy set *A*. Fuzzy set *A* includes all possible candidates or neighbors. The set also has a membership function. The membership function maps each value (neighbor) to a membership value on the range of [0, 1].

Membership function computation: the membership function consists of three factors, *K*1, *K*2, and *K*3, on a range of [0,1]. A fairly efficient way to compute the membership degree of neighbors can be achieved by multiplying these factors together. All three factors of each neighbor will thus be multiplied together to get neighbor's membership degree. Assume there are *n* neighbors. Computing of the factors for the membership function of the *i*th neighbor is described below in more detail:

(3) K1=1−(HopiMaxHop),K2=(EnergyiMaxEnergy),K3=1−(DistanceiMaxDistance).

The membership degree of the *i*th neighbor can then be computed based on the following function and inserted into the *M*_2_ field of the *i*th neighbor:

(4) $$\begin{array}{c} M_{2}=\text{Membership}\;\text{Function}=K_{1}\times K_{2}\times K_{3}. \end{array}$$

#### 3.2.2. Probability Computation

When a node (i.e., node *i*) receives a query packet from a neighbor for the first time (i.e., from neighbor *k*), this produces a new entry in its Neighbor List. The Neighbor List is composed of fields, and each part of the data has to be stored in its related field. The LA can then compute the probability of neighbor *k* from the information contained in the Neighbor List received from neighbor *k* and store it in M1 field of the Neighbor List. The probability *P*_*k*_(*t*) associated with neighbor *k* is computed according to the expression

(5) Pk(t)=13(ℰk(t)∑m=1Niℰm(t)+1/Dk(t)∑m=1Ni(1/Dm(t))   +1/Hk(t)∑m=1Ni(1/Hm(t))) k≤Ni,

where En is the energy level advertised by neighbor *m*, *N* is the size of node *i*'s Neighbor List (including now node *K*), and *D* is the distance advertised by neighbor *m* to the station *S*, computed based on expression (6) where (*x*_1_, *y*_1_) and (*x*_2_, *y*_2_) are the positions of the station and the current neighbor already saved in Neighbor List. The sums in the denominators represent the terms to normalize the probabilities and to make ∑_*k*=1_^*N*_*i*_^ *P*_*k*_(*t*) = 1,

(6) $$\begin{array}{c} D={\left({\left(y_{2}-y_{1}\right)}^{2}+{\left(x_{2}-x_{1}\right)}^{2}\right)}^{1/2}. \end{array}$$

The rationale of using expression (5) is that it produces a good balance between energy and distance, though at the cost of the potential recomputation of the probabilities immediately after each query packet is received, since the sum of the probabilities for all neighbors must be equal to one.

#### 3.2.3. Updating Probabilities

**Figure 3:**
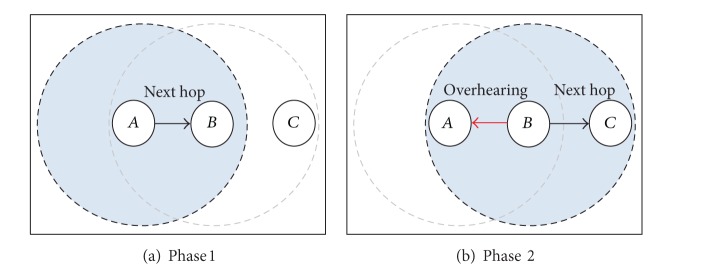
Updated probability scheme.

The basics of the mechanism are illustrated in Figure 3, and it works as follows. The M1:LA module in node *A* offers the neighbor with the highest probability, from the M1 field of Neighbor List, as its offered next hop (neighbor *B* in this case), and then it waits for final decision by the DM algorithm. If the DM selects the same node (node *B* in this case) as a next hop, it informs the LA (learning automata) whose offered neighbor was selected as a next hop.

Thus, in fact, the probability updating will be enabled if the LA and the DM select the same neighbor as the next hop. When node *B* receives the data packet, it updates the piggybacked energy level of node *A* in its Neighbor List and all the other neighbors of *A* overhear the data packet and perform the same updates as *B*, although they discard the packets immediately after processing them. The routing process continues now with node *B* selecting node *C* as its next hop. When *B* sends the data packet to *C*, node *A* is the one that overhears the packet sent by *B*; it thereby updates the energy level of the latter and then it will update probability of node *B*. The updating functions are based on piggybacking and overhearing techniques; they can compute and mutually update the probabilities in the Neighbor Lists according to the energy levels, hop count, and distances obtained from the neighbors. In the example, if the metrics received from node *B* are acceptable, then node *B* is rewarded by the learning automaton in *A*, and the probability associated with *B* is increased in node *A*'s Neighbor List. Otherwise, *B* is penalized and its probability is decreased.

In our model, we considered four behavioral cases for rewarding or penalizing a neighbor *B*.

In the first case, the energy-distance-hop relationship is below the average, and thus the learning automata in *A* will penalize node *B* with a factor *β*, where 〈*ℰ*_*A*_(*t*)〉 = ∑_*m*=1_^*N*_*i*_^ *ℰ*_*m*_(*t*)/*N*_*i*_ represents the average energy of the neighbors of node *A*, and *ℰ*_*B*_(*t*) stands for the energy level obtained from *B*. Likewise, 〈*D*_*A*_(*t*)〉 represents the average distance of the neighbors of *A* to the station *S*, while *D*_*B*_(*t*) represents the distance to *S* reported by node *B*. 〈*H*_*A*_(*t*)〉 represents the average hop count of the neighbors of *A* to the station *S*, while *H*_*B*_(*t*) represents the hop count to *S* reported by node *B*. In the second case, we consider a lower penalization. The penalization selected is *β*/2. In the third case, node *A* will reward node *B* with *α*/2. We consider that the best case occurs in the fourth case.

(7)  Case  1:  ℰB(t)〈ℰA(t)〉+〈DA(t)〉DB(t)+〈HA(t)〉HB(t)<3, Case  2:  ℰB(t)〈ℰA(t)〉+〈DA(t)〉DB(t)+〈HA(t)〉HB(t)=3. Case  3:  3<ℰB(t)〈ℰA(t)〉+〈DA(t)〉DB(t)+〈HA(t)〉HB(t)≤3.5. Case  4:  3.5<ℰB(t)〈ℰA(t)〉+〈DA(t)〉DB(t)+〈HA(t)〉HB(t).

*Reward Computation*. The reward parameter *α* is used during the update mechanism in order to grant more priority to the nodes giving them more possibilities to forward the response packets to the station. The value of *α* is computed using

(8) $$\begin{array}{c} \alpha =\lambda_{\alpha }+\delta_{\alpha }\left(\frac{{ℰ}_{B}\left(t\right)}{\langle {ℰ}_{A}\left(t\right)\rangle }\times \frac{\langle D_{A}\left(t\right)\rangle }{D_{B}\left(t\right)}\times \frac{\langle H_{A}\left(t\right)\rangle }{H_{B}\left(t\right)}\right), \end{array}$$

where *λ*_*α*_ is the minimum reward granted to a well-positioned node, and *δ*_*α*_ is the limiting factor for the reward.

*Penalty Computation*. Similarly, we use

(9) $$\begin{array}{c} \beta =\lambda_{\beta }+\delta_{\beta }{\left(\frac{{ℰ}_{B}\left(t\right)}{\langle {ℰ}_{A}\left(t\right)\rangle }\times \frac{\langle D_{A}\left(t\right)\rangle }{D_{B}\left(t\right)}\times \frac{\langle H_{A}\left(t\right)\rangle }{H_{B}\left(t\right)}\right)}^{-1}, \end{array}$$

where, analogously to the reward mechanism, *λ*_*β*_ is the minimum penalty, and *δ*_*β*_ is the limiting factor. Note that, in expression (8) and (9), the better (or worse) the energy-distance relationship, the greater the reward (or penalization) assigned to node *B*. Upon obtaining the energy and distance metrics from node *B*, the learning automata in node *A* will update the probabilities of its *N*_*A*_ neighbors based on (3) and (11). The former applies to the rewarding cases, that is, the third and fourth cases described above, with *x*_*α*_ = *α*/2 and *x*_*α*_ = *α*, respectively. The latter corresponds to the penalization cases, that is, the first and second cases, with *x*_*β*_ = *β* and *x*_*β*_ = *β*/2, respectively,

(10) $$\begin{array}{c} P_{B}\left(t_{n+1}\right)=P_{B}\left(t_{n}\right)+x_{\alpha }\left[1-P_{B}\left(t_{n}\right)\right],\\ P_{k}\left(t_{n+1}\right)=\left(1-x_{\alpha }\right)P_{k}\left(t_{n}\right)\;\forall k\;|\;k\neq B\wedge k\leq N_{A}, \end{array}$$

(11) PB(tn+1)=(1−xβ)PB(tn),Pk(tn+1)=xβNA−1+(1−xβ)Pk(tn)      ∀k ∣ k≠B∧k≤NA.

## 4. Performance Evaluation

In this section, we evaluate the ERP's performance by comparing it to the following routing protocols: Rumor [5], as a basic query-based routing protocol, and EQR [9], as a new query-based routing protocol. To this end, we used the GloMoSim simulator developed by UCLA [12]. The simulation model used and the results we obtained with it are described below.

### 4.1. Simulation Model

We used a surface that was 1000 m × 1000 m. The radio range was set to 177 m, with an available bandwidth of 2 Mbps and a radio transmission (TX) power of 4 dBm. Each simulation had a 4-hour duration, and the tests were run under various conditions, such as with different amount of sensors, namely, 1000, 1200, and 1400 nodes, and also with 10 different amounts of seeds. Moreover, the placement of the sensors in the terrain and their initial energy levels were selected randomly. It is worth highlighting that even though the placement and initial energy of the nodes were set randomly, once set, those factors remained fixed for rest of the trials to obtain comparable results across experiments. In the simulations presented here, the traffic in the network is always initiated by a source station *S*, which periodically acquires information from a particular sensor *d*. The sensor *d* moves at a speed of 40 metre/hour. Once the query is received at *d*, the sensor will immediately send back the response to *S* with the requested information.

#### 4.1.1. Scenario I

In this scenario, we assume a critical situation, where the energy levels for transmission mode are very low. Under these conditions, we evaluate the different routing schemes considering three different tests.

*Test 1: Time until the First Node Runs Out of Battery Power*. This test is one of the indicators of the effectiveness of the routing schemes in terms of energy management. In general, those with the capacity to balance the energy consumed should last longer without node failure.

*Test 2: Number of Nodes That Run Out of Battery Power*. This test computes the total number of sensors that fail in each routing scheme during a simulation period of 2 hours, providing an indicator of the capacity of the routing schemes for saving energy.

### 4.2. Scenario II

In this scenario, the energy levels of the sensors are set sufficiently high so as to avoid experiencing node failures during the simulation runtime. Our goal in this case is to compare the fairness in terms of energy consumption. In order to avoid bias in the comparison, we ensure that all the routing schemes transmit the same amount of data and that this occurs without node failures. We carry out three tests to examine how the routing schemes save and manage energy in regular operation mode.

*Test 3: Variance in the Remaining Energy Levels for the Neighbors of Station S*. This test allows us to examine which routing scheme is best at performing energy balancing among the nodes close to the station.

*Test 4: Average Energy Consumption*. This test provides another indicator of which routing scheme is more efficient in managing energy.

### 4.3. Simulation Results

#### 4.3.1. Scenario I

**Figure 4:**
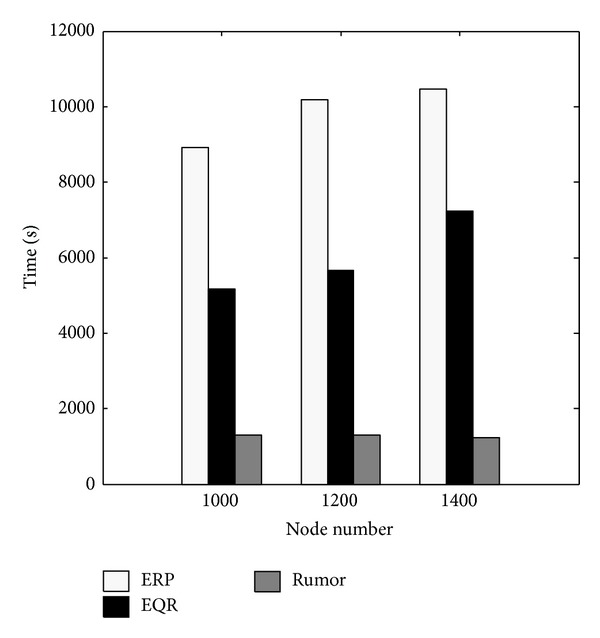
*Test 1:* time until the first node runs out of battery.

The most commonly used measure of network lifetime is the time until the first node runs out of battery energy. In ERP, the first sensor fails after ~8900 to ~10500 seconds depending on the number of nodes present in the network (see Figure 4). The time in which the first node runs out of battery is relatively shorter for EQR and significantly shorter for Rumor.

**Figure 5:**
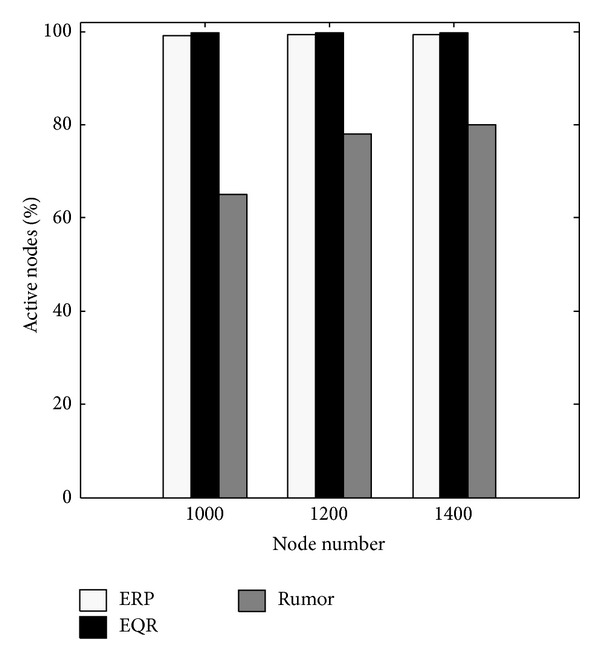
*Test 2:* fraction of active nodes.

Figure 5 shows that the ERP is much better than the more traditional Rumor and that it is relatively similar to the new EQR protocol. In brief, even though in low battery situations there is no big difference between EQR and ERP in terms of the number of node failures, it is clear that ERP can keep a network alive longer than EQR. Its main reason can be related to this point that decision-making system of ERP (LA and FS) is more accurate than EQR (only LA).

#### 4.3.2. Scenario II

**Figure 6:**
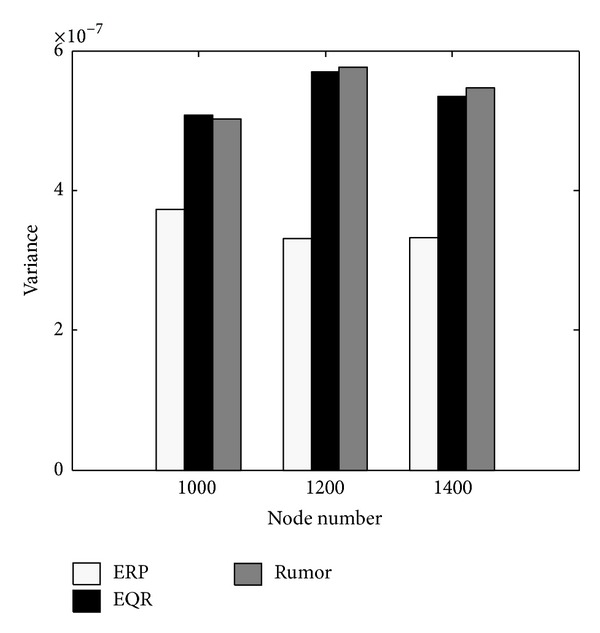
*Test 3:* variance.

**Figure 7:**
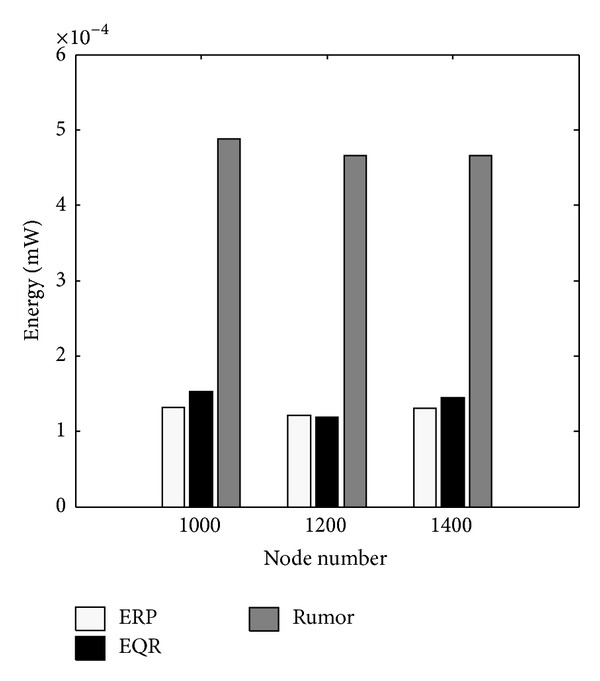
*Test 4:* average energy consumption.

In this scenario we evaluate the quality of these protocols in normal energy situations.  Figure 7 shows that Rumor is the worst routing protocol, mostly because Rumor selects its next hops randomly. There is no visible difference between ERP and EQR in energy consumption, because both protocols use zonal broadcasting. However Figure 6 shows that ERP is more successful and a better energy manager than EQR.

Actually, beyond merely comparing the particular values obtained in each figure, the most important conclusions that can be extracted from the tests as a whole are basically the following. The results show that the combination of a FS technique and LA can improve energy balancing and, more importantly, that the combined operation (ERP's use of fuzzy set plus learning) can work better than using only one technique (EQR).

## 5. Conclusion

In this paper we studied energy-aware query-based routing protocols. From the routing perspective, we have observed that the current destination-initiated query-based routing protocols can be considerably improved, especially, if we aim for a better balance between the energy savings and energy balancing objectives. We have proposed a new energy saver/balancer routing protocol. We simulated and compared our routing protocol with traditional Rumor and newer EQR protocols. Indeed, four different types of tests were carried out and described, and in most of these tests it was indicated that the ERP obtained significantly better results.

## Figures and Tables

**Algorithm 1:**
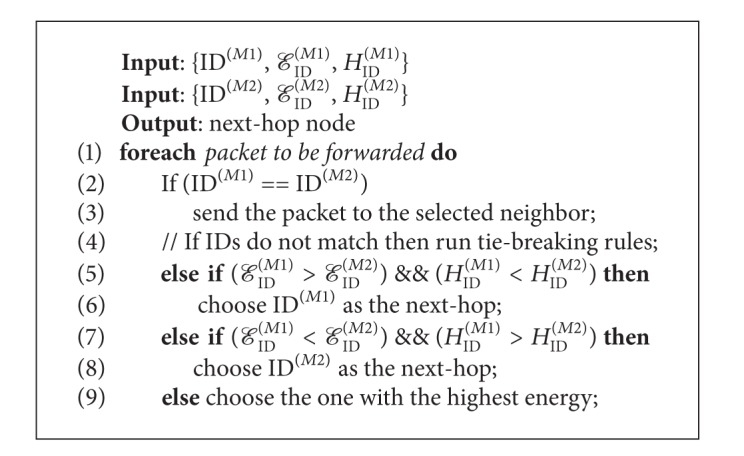
The decision maker (DM) algorithm.
